# Next maSigPro: updating maSigPro bioconductor package for RNA-seq time series

**DOI:** 10.1093/bioinformatics/btu333

**Published:** 2014-06-03

**Authors:** María José Nueda, Sonia Tarazona, Ana Conesa

**Affiliations:** ^1^Statistics and Operational Research Department, University of Alicante, 03690, Alicante, Spain, ^2^Genomics of Gene Expression Laboratory, Prince Felipe Research Centre, 46012 Valencia, Spain and ^3^Applied Statistics, Operational Research and Quality Department, Polytechnic University of Valencia, 46020 Valencia, Spain

## Abstract

**Motivation:** The widespread adoption of RNA-seq to quantitatively measure gene expression has increased the scope of sequencing experimental designs to include time-course experiments. maSigPro is an R package specifically suited for the analysis of time-course gene expression data, which was developed originally for microarrays and hence was limited in its application to count data.

**Results:** We have updated maSigPro to support RNA-seq time series analysis by introducing generalized linear models in the algorithm to support the modeling of count data while maintaining the traditional functionalities of the package. We show a good performance of the maSigPro-GLM method in several simulated time-course scenarios and in a real experimental dataset.

**Availability and implementation:** The package is freely available under the LGPL license from the Bioconductor Web site (http://bioconductor.org).

**Contact:** mj.nueda@ua.es or aconesa@cipf.es

## 1 INTRODUCTION

The use of RNA-seq for transcriptome profiling as a replacement for microarrays has triggered the development of statistical methods to properly deal with the properties of these types of count-based data. RNA-seq measurement of gene expression is based on the number of reads mapped to transcripts, which results in discrete quantities and left-skewed distributions. In contrast, microarray signals are scanned fluorescence intensities, and this translates into continuous and nearly normal expression data. Although normality was typically assumed and linear models (LMs) were applied to model microarray experiments, other distributions such as Poisson and Negative Binomial (NB) capture better the nature of count data. Hence, methods such as edgeR (Robinson *et al.*, 2010) and DEseq (Anders and Huber, 2010) updated microarray analysis to RNA-seq by incorporating appropriate statistical models, whereas other methodologies were developed specifically for the new technology (Roberts and Pachter, 2013; Tarazona *et al.*, 2011; Trapnell *et al.*, 2012). Moreover, sequencing introduces specific biases to gene expression quantitation and, therefore, dedicated normalization methods exist for RNA-seq to correct for sequencing depth, transcript length (Mortazavi *et al.*, 2008), GC content (Risso *et al.*, 2011) and non-uniform transcript distributions (Bullard *et al.*, 2010; Robinson and Oshlack, 2010).

The first RNA-seq experiments were still constrained by the relatively high costs of sequencing in comparison with microarrays, which restricted experimental designs to case–control studies with low replication. As a consequence, the novel statistical methods mostly addressed this analysis scenario. As the technology became more affordable, other types of designs involving more samples, such as time-course experiments, started to appear. In a time-course study, the dynamics of gene expression are evaluated at different time points after induction by a particular treatment or in relation to development. Statistical analysis of time-course data implies the identification of genes that change their expression along time and/or follow a specific expression pattern. maSigPro is an R package designed for the analysis of transcriptomics time courses (Conesa *et al.*, 2006). maSigPro models gene expression by polynomial regression and identifies expression changes along one or across several time series by introducing dummy variables in the model. The method progresses in two regression steps: the first one selects genes with non-flat profiles and the second step creates best regression models for each gene to identify specific time or series-associated changes. The package includes several clustering algorithms and visualization tools to group and display genes with the same expression patterns. maSigPro has been applied in many different biological settings, such as biomedicine (Hoogerwerf *et al.*, 2008), biotechnology (Levin *et al.*, 2007) and plant research (Terol *et al.*, 2007) to cite some, and also has been implemented in several web services (Medina *et al.*, 2010; Nueda *et al.*, 2010) and used in combination with multivariate statistics to analyze multifactorial designs (Nueda *et al.*, 2009) or as batch filtering technique (Nueda *et al.*, 2012). maSigPro was developed to treat continuous microarray intensities and applies LMs to model gene expression. In this article, we describe the update of maSigPro to deal with RNA-seq count data by incorporating generalized linear models (GLMs; Dobson, 2002; McCullagh and Nelder, 1989) into the package and allowing a more flexible choice in the reference family distribution. We demonstrate the appropriateness of this adaptation using simulated and real data and compare the method with edgeR that also accepts time-course designs.

## 2 METHODS

### 2.1 Model

Considering the case of a time-course experiment with *T* time points and *S* experimental groups or series (e.g. different treatments, strains, tissues), maSigPro uses polynomial regression to model the gene expression value *y_i_* at condition *i* and time *t_i_*, and defines *S* − 1 binary variables (*z_s_*) to distinguish between each experimental group and a reference group (Conesa *et al.*, 2006). For the sake of simplicity and illustration of the model, we consider here a quadratic regression and an experiment with two series. The polynomial model of *y_i_* is

$$y_{i}=\beta_{0}+\beta_{1}t_{i}+\beta_{2}t_{i}^{2}+\beta_{3}z_{1i}+\beta_{4}t_{i}z_{1i}+\beta_{5}t_{i}^{2}z_{1i}+\varepsilon_{i}$$

maSigPro originally supported only LMs, where the response variable is modeled as a normal distribution. GLMs are a generalization of classical LMs, which can accommodate a wider class of distributions named as *exponential family*, providing great flexibility for modeling different types of response variables. Normal, Poisson, Binomial, Gamma and NB are examples of this family of distributions. These family classes have generic definitions, which imply that a common maximum likelihood method for estimating the parameters of the model can be applied to all of them. Although explicit mathematical expressions can be found for estimators, iterative numerical methods based on the Newton–Raphson are typically used (Dobson, 2002; McCullagh and Nelder, 1989). In GLMs, hypothesis testing and the goodness of fit of the model are based on the log-likelihood ratio statistic, also denoted as deviance *D*:

$$D=2[l({\hat{\beta }}_{max})-l(\hat{\beta })]∼\chi_{m-p}^{2}$$

where $l({\hat{\beta }}_{max})$ is the maximized likelihood of a model with *m*, the maximum number of parameters that can be estimated, and $l(\hat{\beta })$ denotes the likelihood of the *P*-dimensional parameter β. The difference between the deviance statistics of the model of interest, *M*_1_, and a model without covariates, *M*_0_, is $\Delta =D_{0}-D_{1}∼\chi_{p}^{2}$, which can be used to evaluate the significance of each gene fit. Within the GLMs definition, LMs are recovered when the normal distribution is followed.

To accommodate the GLMs, the existing *p.vector()* and *T.fit()* functions of the maSigPro package that account, respectively, for first and second regression steps of the method have been modified by replacing the function *lm()* by *glm()*. A new argument, denoted *counts*, has been added to select the type of modeling. The default setting is *counts = FALSE* to keep the LMs and by setting *counts = TRUE*, maSigPro will apply the GLMs option with NB distribution. NB is the recommended family to use when dealing with RNA-seq as it allows overdispersion of variance, which is related to the mean through the (θ) parameter:

$$Y_{i}∼NB(\mu_{i},\theta )\text{, where}\;E(Y_{i})=\mu_{i}\;\text{and}\;Var(Y_{i})=\mu_{i}+\frac{\mu_{i}^{2}}{\theta }$$

Theta (θ) can be estimated using available software (for instance edgeR, Robinson *et al.*, 2010). When no estimation of θ is possible, we recommend to use the default value, θ = 10. Our experience indicates that maSigPro results do not change much by using different values of θ. The package also includes the possibility of applying any other available exponential family through the additional argument *family*.

In the second step of maSigPro, the goodness of fit, *R*^2^, of each optimized gene model is computed. This parameter is used for selecting genes with clear expression trends. In LMs, *R*^2^ is defined from the residual sum of squares, and in GLMs the goodness of fit is evaluated in terms of the deviance: the percentage of deviance explained by the model. However, for the sake of consistency with older maSigPro versions, the package maintains the notation *R*^2^ for both LMs and GLMs. The remaining functions of the package stay unchanged.

Note that no explicit normalization procedure is implemented within the maSigPro methodology, and hence, data should be appropriately normalized beforehand. Results presented in this article have been computed by using TMM normalization (Robinson and Oshlack, 2010).

### 2.2 The evaluation strategy

To evaluate the performance of the updated maSigPro to identify differentially expressed genes (DEGs) in RNA-seq time-course data, we have created different synthetic datasets in which we consider several possible experimental designs. Each dataset has been analyzed with maSigPro-LM, maSigPro-GLM and edgeR. Comparison with maSigPro-LM was included to highlight the limitations of this modeling with count data when the number of replicates is low, even after normalization.

Both maSigPro and edgeR methods are based on the GLMs but with a different approach. The major difference between the maSigPro and edgeR methods is that maSigPro is specialized in the estimation of serial data, i.e. when the independent variable is quantitative such as time. This is achieved by providing an easy way to define a polynomial model for the data. Another important difference is that maSigPro follows a second stepwise regression that obtains the best model for each gene and retains only significant coefficients in each model, whereas edgeR applies the same model to each gene.

#### 2.2.1 Simulated data

Simulations have been created using NB distributions with a parametrization based on the mean μ and size θ. In each sample *i*, where the targeted total number of reads is *N*, and the relative abundance of each gene *g* is *p*_*gi*_, the expected gene counts, μ_*gi*_, can be computed as

$$\mu_{gi}=N\times p_{gi}$$

Note that, as gene counts are randomly drawn from a NB distribution, the simulated count values of each gene will slightly vary among samples and so will the total number of reads *N_i_* of the sample *i*.

Simulated datasets were designed to contain genes that belong to one of the *K* = 4 gene expression level classes, which are defined by a fixed reference value at time 1 (*v_k_*_1_) and a given size (*n_k_*, number of genes) in each *k* level as indicated in Table 1.

**Table 1.** Reference *v_k_*_1_ values for K = 4 groups

Expression	Reference value *v_k_*_1_	Number of genes *n_k_*	Genes (%)
Low	5	10 000	50
Median	50	8000	40
High	500	1900	9.5
Very high	5000	100	0.5
		20 000	100

To model time-associated gene expression changes we considered the following linear expression:

$$\begin{array}{ccc} v_{gi}=v_{k1}+b_{g}v_{k1}t_{i}, & \{\begin{array}{cc} b_{g}=0, & \text{if}\;g\;\text{is not DEG}\\ b_{g}\neq 0 & \text{if}\;g\;\text{is DEG} \end{array} & \;i=2,\ldots ,T \end{array}$$

where 5% genes have *b_g_* values different from zero and are differentially expressed. Furthermore, we modeled three different data scenarios by assigning different values to the *b_g_* parameter to subsets of genes: (A) In this scenario, all DEGs increase their expression linearly with *b_g_* = 0.2; (B) In this scenario, half of the DEGs increase *b_g_* = 0.2 and half decrease with $b_{g}=-0.2$, and we added, when needed, a positive value to *v_g_*_1_ to avoid negative means; (C) Genes follow a strong upregulation in the second time-point followed by decrease with $b_{g}=-0.2$.

Datasets were modeled either with one or two time series. In the two series case, one series was modeled as described and the second was modeled as a flat profile. For each scenario and series number, datasets were simulated with 1, 2, 3 or 5 replicates. Finally, genes were considered to have constant length equal to 1 kb in all datasets and no length correction was applied in the data.

Following this simulation scheme, the relative proportion of counts of gene *g* in sample *i* is

$$p_{gi}=v_{gi}/\sum_{g}\left(v_{gi}\right)$$

This approach provides the way to take into account not only the expression level, but also the composition of the RNA population in the sample, as gene proportions are computed a posteriori and are affected by the gene expression changes modeled in each scenario.

#### 2.2.2 Experimental data

The maSigPro-GLM and compared methods were evaluated on a real dataset that describes the transcriptional response of inmunocompromised *Arabidopsis thaliana* lines to the barley powdery mildew fungus *Blumeria graminis* (Bgh) (Hacquard *et al.*, 2013; Maekawa *et al.*, 2012). In this study, pen2 pad4 sag101 Arabidopsis plants harboring (pps) or without (B12) the MLA1-HA construct were challenged with either the Bgh isolate K1 expressing the cognate AVRA1 effector for MLA1 or the Bgh isolate A6 expressing other AVRA effectors. Three independent biological replicates per condition were harvested at 6, 12, 18, 24 h post-inoculation. The experimental design of this study has therefore 4 time points, 2 covariates with 2 levels each one: MLA1 (pps or B12) and Bgh isolate (A6 or K1), 3 replicates and 6477 genes. Initial analysis of these data revealed little effect of the MLA1 construct covariate, which was then eliminated from the model for simplicity. Therefore, in the maSigPro formulation, this experiment corresponds to a replicated 4 time points course with two series (Bgh isolate A6 or K1). Data are available at http://www.ncbi.nlm.nih.gov/geo/query/acc.cgi?acc=GSE43163.

## 3 RESULTS

### 3.1 Simulation studies

The simulation experiment contained 24 datasets obtained by combining three secenarios (A, B and C), one or two time series and one of the four replication levels. Datasets were created with θ = 10, and 6 time points. Here, we show results from data with 20 000 genes. Simulations with a smaller dataset of 6000 genes gave similar results.

**Fig. 1.**
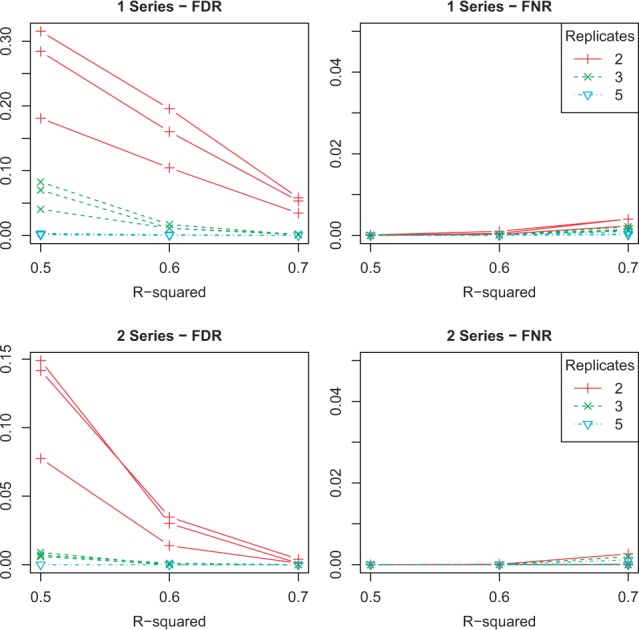
FDR and FNR for maSigPro-GLM at different levels of *R*^2^ with 1 and 2 series

One of the challenges in the development of the maSigPro-GLM methodology was to establish an appropriate cutoff value for the *R*^2^ parameter in the second regression step. We analyzed False Discovery Rate [FDR : false positives (FP)/Selection] and False Non-discovery Rate [FNR: false negatives (FN)/Non-selected] for varying *R*^2^ values at fixed FDR = 0.05 (Fig. 1). We observed that as the number of replicates increase, FDR and FNR drop and that the two series scenario is slightly better than the one series case. In general, for *R*^2 ^= 0.7 the method achieves a good control of FDR with negligible FNR. However, in designs with three replicates and two series, and when five replicates are available, FDR is also controlled by *R*^2 ^= 0.5. Taking this result into account, we applied a *R*^2 ^= 0.7 cutoff value to obtain performance metrics in our simulation study. Table 2 shows the number of selected genes, FP and FN for the three methods at a FDR = 0.05. Several conclusions can be drawn from these results:

1. Absence of replication is clearly insufficient for appropriate time-course modeling. maSigPro-LM is unable to find DEGs and maSigPro-GLM calls too many FP. edgeR is not recommended for unreplicated data and, therefore, not used in this case.
2. In general, maSigPro-LM performs poorly on RNA-seq data in all scenarios and conditions.
3. Given two or more replicates, maSigPro-GLM succeeds in controlling FDR <5%, whereas edgeR tends to give moreFP, ranging between 11 and 20% false calls.
4. FNR is properly controlled both by maSigPro-GLM and edgeR. This last method has a zero false call rate in our simulations, whereas maSigPro-GLM shows FNR <1%. Results were basically similar considering one or two series and different expression patterns for DEGs.

**Table 2.** Simulated experiments results with scenarios A, B and C for maSigPro-LM, maSigPro-GLM and edgeR

(Scenario) # Series	Rep	maSigPro-LM			maSigPro-GLM			edgeR		
		Sel	FP	FN	Sel	FP	FN	Sel	FP	FN
(A)										
1 Series	1	1	0	999	2210	1496	286			
	2	533	25	492	976	52	76	1135	135	0
	3	589	5	416	975	2	27	1173	173	0
	5	515	0	485	997	0	3	1170	170	0
	
2 Series	1	471	34	563	1969	972	3			
	2	981	5	24	1001	1	0	1267	267	0
	3	985	1	16	1000	0	0	1278	278	0
	5	995	0	5	1000	0	0	1219	219	0
(B)										
1 Series	1	0	0	1000	1592	741	149			
	2	723	46	323	990	34	44	1158	158	0
	3	750	2	252	978	1	23	1155	155	0
	5	751	0	249	994	0	6	1136	136	0
	
2 Series	1	253	14	761	1351	411	60			
	2	672	4	332	951	1	50	1240	240	0
	3	592	0	408	963	0	37	1225	225	0
	5	538	0	462	978	0	22	1138	138	0
(C)										
1 Series	1	0	0	1000	1427	764	337			
	2	284	14	730	972	37	65	1166	166	0
	3	433	3	570	945	0	55	1125	125	0
	5	357	0	643	963	0	37	1134	134	0
	
2 Series	1	222	12	790	1458	471	13			
	2	684	9	325	996	2	6	1284	284	0
	3	378	0	322	999	0	1	1201	201	0
	5	681	0	319	998	0	2	1209	209	0

*Note*: Number of replicates (Rep), number of selected genes (Sel), false positives (FP) and false negatives (FN).

### 3.2 Experimental study

**Fig. 2.**
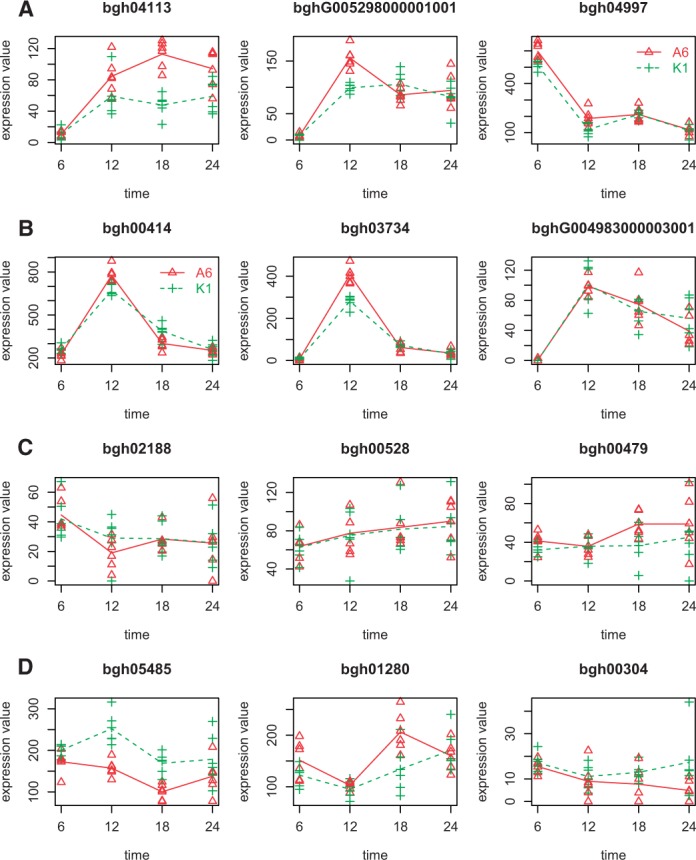
Random examples from genes selected with (**A**) maSigPro and edgeR, (**B**) maSigPro and not with edgeR, (**C**) with edgeR and not preselected with maSigPro and (**D**) with edgeR and not with maSigPro because *R*^2 ^< 0.5

We applied both edgeR and maSigPro-GLM to the *A.thaliana* time-course data considering the two series defined by the Bgh isolate. An *R*^2^ threshold of 0.5 was chosen for the second maSigPro-GLM step, according to the results presented in Figure 1. Genes with <100 reads in all samples were discarded, resulting in a dataset containing 5838 genes. edgeR identified 2870 DEGs across the different time points, whereas maSigPro-GLM selected 2158 DEGs (FDR = 0.05). There were 1629 genes in common between the two methods, 529 specifically found by maSigPro and 1241 identified only by edgeR. Out of these 1241 edgeR exclusive DEGs, 1194 were identified as significant in the first maSigPro but finally not selected in the second regression step because their *R*^2 ^< 0.5, while the remaining 47 genes were not preselected by maSigPro in the first step. To better understand the gene expression patterns associated to similarities and differences between the two methods, we randomly selected three genes belonging to each of these sets (Fig. 2). These examples suggested that genes selected by both methodologies and exclusively by maSigPro (A and B) have good regression models, clean expression trends and strong expression changes. Genes selected by edgeR and not preselected by maSigPro (C) show little fold change and high variance, and genes that edgeR calls significant but do not pass the second regression step in maSigPro (D) used to display time-point-specific variances and expression differences.

## 4 DISCUSSION

In this work, we describe and justify the modifications introduced in the maSigPro package to deal with RNA-seq data. We have incorporated GLMs into the first and second regression steps of the algorithm and add the parameter *counts* into the *p.vector()* function to select the type of statistical modeling. Setting *counts = TRUE* chooses the GLMs and applies the NB distribution, whereas *counts = FALSE* selects the Linear Model as previously. The remaining functions for defining the polynomial model, selecting genes, clustering and visualization remained unchanged, making maSigPro a unified package for the analysis of both microarray and RNA-seq time-course data.

maSigPro applies GLMs to model RNA-seq as do other dedicated statistical packages such as edgeR, included for comparison in this study. The major difference between maSigPro and edegR methods is that maSigPro is specialized in parameter estimation of serial data, i.e. when the independent variable is quantitative such as time. This is achieved by providing an easy way to define a polynomial model for the data that have the flexibility to fit different time-course patterns. In contrast, edgeR treats time not as a continuous variable but as multifactor. Another important difference is that maSigPro follows a second step that obtains the best model for each gene such that only significant coefficients are retained in each model, whereas edgeR applies the same model to each gene under the multifactor consideration. This results in models with more variables that might be prone to give false calls. Moreover, we apply in the second step a filter on gene selection that takes into account the *R*^2^ of the regression model, implying that only genes with a good fit to the model will be selected. The consequences of the different implementations are clear in the results of the simulation study and the experimental data. Basically, we observed a better control of FDRs in maSigPro and that genes selected by maSigPro have not only significant models but also well-fitted models. Finally, the maSigPro package also provides clustering and visualization of significant genes.

One important aspect that we considered in our simulation study was the number of replicates and the complexity of the time-course experiment (one or two comparing series). Our results indicate that one replicate is clearly not sufficient for the proper control of the FDRs. While initial RNA-seq took advantage of the accuracy of the technology to avoid replication, recent studies highlight the importance of appropriate replication for a sound RNA-seq data analysis (Liu *et al.*, 2014; Sims *et al.*, 2014; Tarazona *et al.*, 2011). Within the parameter settings of the simulation experiment, we show that maSigPro-GLMs controls FDR and FN from two replicates and that the performance improves as the number of replicates and series increase. Related to this, it is also interesting to comment results of the maSigPro-LM analysis on the synthetic data. While it might be obvious that LMs are not appropriate to model count data, one could speculate that after data normalization, discretization would be removed and the normalized data could be treated as continuous data. However, transformed data are not normally distributed, and right asymmetry still holds. Although transformed data do not necessarily conserve the probability distribution of the untransformed data, the GLMs fitting process mainly depends on the assumed variance-to-mean relationship. Linear transformations of the data do not change these relations and link functions such as the logarithm are not exclusive for discrete data. This becomes evident when looking into the maSigPro-LM results on the simulated data: the linear model performs poorly in most scenarios. However, the central limit theorem suggests that models developed for normal data can be applied to non-normal data if the available sample is large enough. We show that maSigPro-LM can achieve good FDR control when five replicates per condition are used in the two series scenario, although still suffering from a significant rate of false-negative calls. The versatility of the maSigPro package to choose the LMs or GLMs with one simple argument option allows easy adaptation of the methodology to the types of data and experimental design.

Finally, although significance thresholds in maSigPro-GLM maintain their statistical meaning, the goodness of fit, which is used in the second step of maSigPro to select genes with well-fitted models, is evaluated in GLMs in terms of the deviance: the percentage of deviance explained by the model. We conducted experiments with simulated data to understand how this parameter behaves in different experimental settings. Our results indicated that similar to the recommended threshold in the LM version of maSigPro, a cutoff value of 0.7 is valid in most scenarios. However, when data are abundant, i.e. triplicated measurements and multiple series, this threshold could be lowered to 0.5. Indeed, this value was used in the analysis of the real Arabidopsis dataset. The comparison with edgeR, which solely selects genes on the basis of a significant *P*-value, showed that the maSigPro filtering based on a *R*^2^ cutoff value resulted in genes with consistent models. Genes that were significant with both methods but discarded by maSigPro because of a *R*^2 ^< 0.5 used to have outliers or highly variable measurements (Fig. 2).

In conclusion, we show that maSigPro-GLM is suitable for the identification of DEGs from time-course RNA-seq data under a wide range of experimental settings. The updated package successfully controls both false-positive and false-negative detection rates.

*Funding*: This work has been funded by the FP7 STATegra [GA-30600] project, EU FP7 [30600] and the Spanish MINECO [BIO2012-40244].

*Conflicts of Interest*: none declared.
